# The Nature and Persistence of Posthypnotic Suggestions' Effects on Food Preferences: An Online Study

**DOI:** 10.3389/fnut.2022.859656

**Published:** 2022-05-04

**Authors:** Anoushiravan Zahedi, Renin Öznur Akalin, Johanna E. Lawrence, Annika Baumann, Werner Sommer

**Affiliations:** ^1^Department of Psychology, Humboldt Universität zu Berlin, Berlin, Germany; ^2^Department of Decision Neuroscience and Nutrition, German Institute of Human Nutrition (DIfE), Nuthetal, Germany; ^3^Neuroscience Research Center, Charité-Universitätsmedizin Berlin, Corporate Member of Freie Universität Berlin, Humboldt-Universität zu Berlin, and Berlin Institute of Health, Neuroscience Research Center, Berlin, Germany; ^4^Berlin School of Mind and Brain, Humboldt Universitat zu Berlin, Berlin, Germany; ^5^Weizenbaum Institute for the Networked Society, Berlin, Germany; ^6^University of Potsdam, Chair for Business Informatics, esp. Social Media and Society, Potsdam, Germany

**Keywords:** eating behavior, food choice, food preferences, hypnosis, online-supermarket, posthypnotic suggestions

## Abstract

Food preferences are crucial for diet-related decisions, which substantially impact individual health and global climate. However, the persistence of unfavorable food preferences is a significant obstacle to changing eating behavior. Here we explored the effects of posthypnotic suggestions (PHS) on food-related decisions by measuring food choices, subjective ratings, and indifference points. In Session 1, demographic data and hypnotic susceptibility of participants were assessed. In Session 2, following hypnosis induction, PHS aiming to increase the desirability of healthy food was delivered. Afterward, a task set was administrated twice, once when PHS was activated and once deactivated. The order of PHS activation was counterbalanced across participants. The task set included a liking-rating task for 170 pictures of different food items, followed by an online supermarket where participants were instructed to select enough food for a fictitious week of quarantining from the same item pool. After 1 week, Session 3 repeated Session 2 without hypnosis induction in order to assess the persistence of PHS. The crucial dependent measures were food choices, subjective ratings, and the indifference points as a function of time and PHS condition.

## Introduction

The increasingly obesogenic prevalent diets (1, 2) in modern society (e.g., high in sugar or salt, high-fat red meat, ultra-processed food, “junk food”) are posing threats to human health, biodiversity, and the climate. Therefore, there is an urgent need to shift toward more healthy diets [e.g., (3)]. The rampant obesity epidemic demonstrates that traditional efforts toward diet change are insufficient (4–7). Therefore, it is crucial to seek new ways to strengthen healthy food choices. Notably, food choices are subject to several interacting factors: food preferences, impulsive reactions, and cognitive control (8–11). Often, good intentions to eat healthy food disintegrate under the force of competing preferences or impulsive behavior. The traditional approach to diet regulations focuses mainly on unhealthy food restrictions through strengthening cognitive control, which showed limited success at best [for review, see (12, 13)]. In the present study, we explore the utility of posthypnotic suggestions (PHS) in biasing food preferences in favor of a healthier diet.

Improving diet habits, which are formed already during sensitive periods early in life (14, 15), requires increasing the preference for and desirability of healthy food on an affective level (16). The acquisition and modulation of food preferences and eating habits involve congenital factors, exposure (17), and a multitude of cognitive (13), affective (16), social, and cultural influences (18) that no single intervention can shoulder. However, PHSs can integrate cognitive and psychosocial factors and successfully change implicit food preferences toward more healthy options (16, 19). Nevertheless, previous efforts were (1) mainly focused on food preferences and not on actual food choices, (2) did not investigate the persistence of the effects, and (3) only recruited participants who were at least moderately responsive to hypnotic suggestions. These issues are addressed in the present study.

To better estimate the effects of PHSs in real-life-like situations, we utilized (I) an online shopping mockup that included a large number of food items and (II) also measured subjective values for the same items. By measuring both subjective values and food choices, we were able to calculate indifference points of food items. Indifference points in binary choices refer to positions where agents might accept or reject an item with a similar probability (20, 21), which can be used to shed light on the underlying cognitive mechanisms of choice behavior. Additionally, in order to address whether the effects persist over time, we re-tested the effects of PHSs after 1 week. Finally, to assess the generalizability of the previous results (16), we recruited participants regardless of their responsiveness to hypnotic suggestions.

### Hypothesis

Together, food choices, preferences, and indifference points can be used to elucidate the mechanisms underlying PHS effects. If choices and preferences for low-calorie food items are increased in the PHS-activated compared to the PHS-deactivated condition, but indifference points are unaffected, one can conclude that PHS modulates choices by affecting explicit preferences. On the other hand, if choices of low-calorie food items are increased, but preferences are not, then a decrease in indifference point may indicate that PHS affects implicit food preferences that are not explicitly accessible. Finally, the increase in preferences without any modulation of choices, but accompanied by increased indifference points for low-calorie items, will indicate that PHS can only affect explicit preferences that are insufficient for affecting choices.

Concerning high-calorie food items, if preferences and choices are decreased, stable indifference points indicate that PHS modulates choices though affecting explicit food preferences. In contrast, if choices of high-calorie food items are reduced but not preferences, an increase in indifference points should be expected. This can be interpreted as related to an increased contribution of top-down cognitive control in food choices. Notably, for high-calorie food items, we do not expect a decrease in preferences that is not accompanied by decreased choices.

Furthermore, we expect the PHS effects on food choice and food preferences to be stable across sessions. Finally, we expected that in both sessions, participants' hypnotizability would be correlated with the observed behavioral effects.

## Methods

### Sample Size and Inclusion Criteria

About 40 participants would be recruited *via* different media. The sample size was chosen based on a priori power analysis with $\alpha <0.05,\;1-\beta >0.95,\;\eta_{p}^{2}>0.08$. The critical values are determined based on the suggestion of Cohen (22), and the effect size is estimated based on previous results [e.g., (16)]. Notably, Zahedi et al. (16) found a medium effect size of $\eta_{p}^{2}\;=\;0.22$. However, since only medium- and high-hypnotizable participants were included in that sample, we adjusted the expected effect size for the current study from medium, i.e., $\eta_{p}^{2}\;=\;0.22$, to small, i.e., $\eta_{p}^{2}\;=\;0.08$. This adjustment is crucial as in the current study, participants are included regardless of their hypnotizability scores, and therefore, we will also have low- in addition to medium- and high-hypnotizable participants.

After first contact, we excluded underweight (BMI <18) and obese (BMI > 30) individuals and anyone with a history of psychological or neurological problems. Individuals outside of the acceptable BMI range would be informed about the reasons for their exclusion and the recommended range of healthy body weight (i.e., BMI = 18–30) and are advised to consider contacting their physician. The study was approved by the ethics committee of the Institut für Psychologie of the Humboldt Universität zu Berlin (approval number 2021-36). Prior to the experiment, informed consent is obtained according to the declaration of Helsinki, and participation is compensated by money (i.e., 10 euro/h) or course credit. The study is conducted fully online.

### Materials and Tasks

The hypnotizability of participants is measured by the German version (23) of the Harvard group scale of hypnotic susceptibility—form A [HGSHS: A (24)]. In HGSHS: A, 12 different suggestions are delivered to participants, and their responsiveness is determined based on the number of items to which they could objectively (based on a self-report questionnaire) respond. Each participant can achieve a score between 0 and 12 according to the scoring procedure suggested by Kihlstrom and Register (25).

Other materials consisted of the Edinburgh Handedness Questionnaire (26), the German Nutrition Knowledge Questionnaire (NKQ) (27), and the Self-Regulation of Eating Behavior Questionnaire (SREBQ) (28). The NKQ consists of 22 questions about the knowledge of healthy food choices and the sources of nutrients in food. The SREBQ consists of four questions aiming to evaluate an individual's capacity for regulating eating behavior.

**Figure 1:**
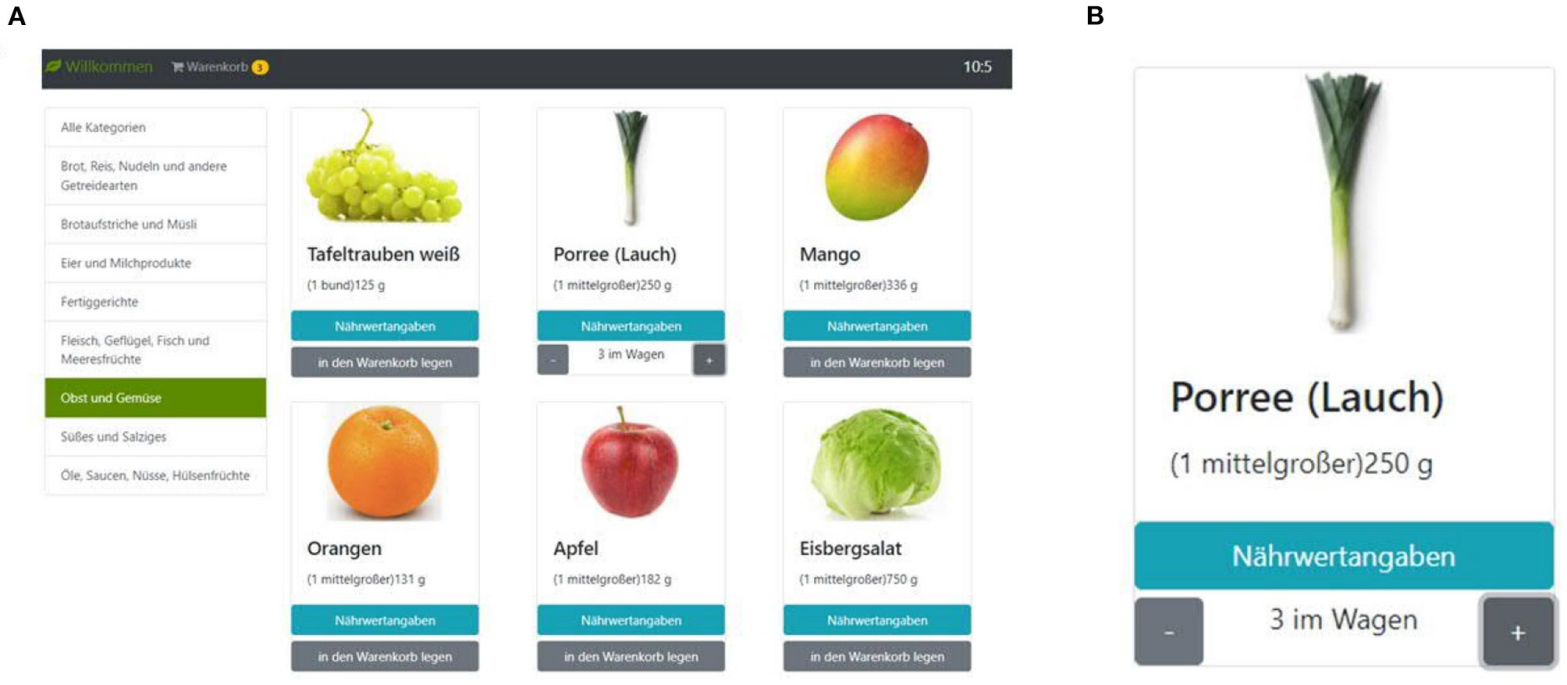
The screenshot of the supermarket task. **(A)** Participants can choose different food categories shown on the left side to see their items. On the top right corner, the remaining shopping time is presented, and on the left top corner, they can see their shopping basket and modify its content. **(B)** For each object, participants can see the nutritional values and select how many they wish to put in their basket.

The online supermarket (Figure 1) was organized based on eight food categories, including 170 products in total. The items were inspired by existing online shops in Germany aiming to simulate near-real life food shopping behavior. For instance, a diverse array of options was presented for each product (i.e., full-fat milk and low-fat milk) to enable participants to choose their preferred items.

The eight categories of food items in the supermarket are as follows:

1: Bread, rice, pasta, and other grain products (e.g., toast bread, pretzel, croissant, etc.),
2: Bread spreads and breakfast cereals (e.g., honey, jams, chocolate creams),
3: Eggs and dairy (e.g., milk, cheese, yogurt),
4: Convenience foods (e.g., filled pasta, pizza, potato salad),
5: Meat, poultry, fish, seafood (e.g., salami, minced meat, smoked salmon),
6: Fruits and vegetables (e.g., tomato, onion, pepper),
7: Sweets and salty snacks (e.g., chocolate, candy, ice cream),
8: Oils, sauces, nuts, legumes (e.g., olive oil, cashew nuts, ketchup).

After choosing a category, between 16 and 26 images per category are shown. For each item, its name is shown above the image; further, the nutrition facts of each item can be inspected by clicking a corresponding button on the screen. For each item, the package size is relatively small to be equal to approximately one average serving size; for example, participants can choose to buy a single egg or a single potato. However, there is no limit to the number of a given item that can be placed in the shopping basket. Each item can be placed in the shopping basket by pressing a corresponding button on the screen. Also, participants can directly select a specific number (i.e., 1 < *n* <20) of each item. The shopping basket can be inspected as well, and the number of items can be corrected before making the final decision. The online supermarket is conducted with the instructions that participants should imagine that they have to quarantine for ~1 week. They should order all food they want to consume during this period from the online supermarket. They have no budget limit and can choose as many products as desired. The only restriction is that the time limit for the online supermarket task is 15 min in total. We did not introduce budgetary restrictions into the online supermarket task because it might interact with or even overshadow the effects of participants' preferences on their food choices [e.g., (29–31)]. This question, however, might be tackled in subsequent research after demonstrating that PHS can indeed affect food choices.

In the food preference rating task, participants are shown all the food items offered in the supermarket task in randomized order. Participants are to rate each item for how much they like it and independent of whether they want it at the moment. Ratings are performed on a Likert scale from 1 (Don't like it at all) to 7 (Like it very much). There is a response window of 5 s for each item, after which the trial is considered a miss. The food preference rating task requires about 10 min.

### Procedure

The experiment is conducted online *via* Zoom (or a similar) platform and involves three sessions. All questionnaires are implemented through the SoSci Survey platform (Version 3.0. 01, www.soscisurvey.de), and the individualized links are sent to participants in real-time during each session. In Session 1, written informed consent is obtained, and demographic information (age, sex, height, weight, educational background), NKQ, and SREBQ are collected. Afterward, the German version of HGSHS: A is administered in order to determine the hypnotic susceptibility of participants. We do not exclude any participants based on the screening results. Instead, susceptibility scores were used as a regressor in other analyses. Session 1 takes about 2 h and is conducted as an online group session (with up to five participants).

About 1 week after Session 1, Session 2 is conducted, lasting about 2 h. In Sessions 2 and 3, each participant is tested individually. Session 2 starts with hypnosis that includes a PHS aiming to induce a strong desire for healthy food. The hypnosis procedure and the employed PHS are the same as in Zahedi et al. (16). Next, the food preference rating and the online supermarket are conducted twice, once with the posthypnotic suggestion activated and once deactivated. The order of conditions (i.e., posthypnotic suggestion activated and deactivated) will be counterbalanced across participants.

Session 3, following between 3 and 10 days after Session 2, is identical in its procedure to Session 2, except that hypnosis is not applied again. The order of PHS activation and deactivation for each participant is the same as in Session 2. The interval between Session 2 and Session 3 appears to be justified considering the effects of PHS in other contexts. For instance, Böhmer and Schmidt (32) have shown that a safety-promoting PHS was effective even over several weeks (Median = 49 days, Range = 7–169 days) after the hypnosis induction.

### Data Analysis

Based on our previous results (16), we expect that posthypnotic suggestions will increase subjective preferences for healthy low-calorie food items and decrease the subjective preferences for unhealthy high-calorie food items without affecting indifference points of these items. That means participants choose what they want based on the same principles as before, and therefore, their indifference points are unaltered. However, their preferences for healthy food items are increased, and hence, they choose healthier options. Following Clark et al. (33), we categorize as low-calorie healthy items: (1) vegetables, (2) fruits, (3) legumes, and (4) fish and marine products. Unhealthy food items were: (1) red meat, (2) processed and ultra-processed food, and (3) sugary and salty snacks. All remaining food items are categorized as neutral. By conducting general linear modeling, we investigate whether the posthypnotic suggestion condition and its interaction with food categories and time affected either subjective food preferences, as measured by the food rating task, or food choices, as measured by the shopping task.

In each model, the PHS condition (PHS-activated vs. PHS-deactivated), Session (Session 2 vs. Session 3), Healthiness (healthy, neutral, and unhealthy food items), and the interaction between these factors are included as fixed effects. Further, a random intercept for the participants and a random slope for their hypnotizability will be assumed (Equation 1). A model with only a intercept will be compared to the full model described above to gauge whether each factor contributes significantly to the results.


(1) $$\begin{array}{l} \text{Outcome}\;\sim \;\text{PHS}\;*\;\text{Healthiness}\;*\;\text{Session}+\\ \;\left(1+\text{Hypnotizability}|\text{Subjects}\right) \end{array}$$

Further, if any significant behavioral result was observed, we will test the point-biserial correlation between the observed effects and hypnotizability scores.

The results from the food rating and the online supermarket tasks are used to calculate indifference points (20, 21) for all food categories with sufficient responses and per posthypnotic suggestion condition and session. In the shopping task, chosen and non-chosen items are designated as 1 and 0, respectively. Indifference points are calculated using logistic regression modeling. For each participant in each condition, choices were entered into the model as a binary input (i.e., yes = 1, no = 0) and subjective ratings as continuous predictors. The output of the model represents the probability of choosing an item giving the subjective rating for that item:


(2) $$\begin{array}{r} p_{j,i,k}\left(Y\right)=\frac{1}{1+\exp^{\left(\beta_{0}+\beta_{1}x\right)}} \end{array}$$

Where *x* designates subjective rating, *Y* choice, *j* participant number, *i* session (e.g., pre-training), *k* calorie content (e.g., low-calorie), and β_0_ and β_1_ are model parameters. Then for each of the remaining participants at each condition, the indifference points are defined as the subjective rating that predicts choosing an item with the probability of 50%. The indifference points are analyzed with the same approach used for assessing subjective ratings and food choices.

### Exploratory Analyses

In exploratory analyses, we will investigate the correlations between observed effects and NKQ and SREBQ results. Further, as macronutrients (e.g., proteins, carbohydrates, and fats) can affect health and cognition [for review, see (34)], we extracted the macronutrients information of the items in the shopping baskets for each condition and session. First, we assess whether the PHS condition affected the macronutrients balance. Finally, the amounts of macronutrients coming from different food categories are calculated to assess the effects of PHS and time.

## Data Availability Statement

The raw data supporting the conclusions of this article will be made available by the authors, without undue reservation.

## Ethics Statement

The studies involving human participants were reviewed and approved by Ethics Committee of the Department of Psychology of the Humboldt-University. The patients/participants provided their written informed consent to participate in this study.

## Author Contributions

AB: methods development. AZ: conceptualization, design, manuscript writing, methodology, and data analysis. JL and RÖA: data acquisition, data analysis, and manuscript writing. WS: conceptualization, design, and manuscript writing. All authors contributed to the article and approved the submitted version.

## Funding

This research was supported by a grant from the Milton Erickson Gesellschaft für klinische Hypnose e.V. to AZ and WS and also partly funded by the Federal Ministry of Education and Research of Germany (BMBF) under grant no. 16DII127 (Deutsches Internet-Institut).

## Conflict of Interest

The authors declare that the research was conducted in the absence of any commercial or financial relationships that could be construed as a potential conflict of interest.

## Publisher's Note

All claims expressed in this article are solely those of the authors and do not necessarily represent those of their affiliated organizations, or those of the publisher, the editors and the reviewers. Any product that may be evaluated in this article, or claim that may be made by its manufacturer, is not guaranteed or endorsed by the publisher.
